# Early treatment for benign paroxysmal positional vertigo secondary to sudden sensorineural hearing loss

**DOI:** 10.1097/MD.0000000000035480

**Published:** 2023-10-06

**Authors:** Gui Fang Li, Man Liu, Yan Zhuo Zhang, Yue Tang Wang, Lan Su, Ran Ran Liu

**Affiliations:** a Department of Otolaryngology, Hebei Provincial Eye Hospital, Hebei Provincial Key Laboratory of Ophthalmology, Hebei Provincial Eye Institute, Xingtai, Hebei, China.

**Keywords:** benign paroxysmal positional vertigo, early, immediate effect, reduction, sudden sensorineural hearing loss

## Abstract

Sudden sensorineural hearing loss (SSNHL) accompanied by benign paroxysmal positional vertigo (BPPV) is relatively common in the clinic. There are unified standards for the treatment of primary BPPV with good reduction effect, while there are few studies on the treatment of BPPV secondary to SSNHL within 1 week of onset. The study was to investigate the treatment of BPPV secondary to SSNHL and compare its manual reduction with that of primary BPPV. We selected 90 patients with BPPV accompanied by SSNHL within a week of onset and 210 primary BPPV patients at Hebei Provincial Eye Hospital from June 2020 to December 2022. The former group was divided into the medicine group and manual reduction plus medicine group. The medicines used were extract of Ginkgo biloba leaves injection, betahistine hydrochloride injection and oral prednisone. We contrasted the efficacy respectively for posterior semicircular canal BPPV (psc-BPPV), horizontal semicircular canal BPPV (hsc-BPPV) and multiple semicircular canal BPPV (msc-BPPV). In addition, we compared the manual reduction effect for primary BPPV and manual reduction group, and the evaluation of efficacy are the intensity of nystagmus and the clinical symptoms. In the secondary BPPV group, there was no difference in efficacy between the medicine group and manual reduction group at the 7th-day after reduction for psc-BPPV, hsc-BPPV, and msc-BPPV (*P* > .05). The immediate effect of reduction was significantly different between the primary BPPV group and the group with SSNHL and BPPV for both psc-BPPV and hsc-BPPV (*P* < .05), and the effect of the primary BPPV group was better, but it was no difference for msc-BPPV (*P* > .05). For the treatment of BPPV accompanied by SSNHL within 1 week of onset, the additional reduction therapy showed no benefit, so we need to apply medication for SSNHL.

## 1. Introduction

Sudden sensorineural hearing loss (SSNHL) is a common ear disease with an incidence of 8 to 10/100,000,^[1,2]^ among which some patients may develop secondary benign paroxysmal positional vertigo (BPPV).^[3]^ BPPV is a temporary vertigo induced by moving the head to a specific position. It is a self-limited, peripheral, vestibular disease and can be divided into primary and secondary types.^[4]^

BPPV is the most common cause of recurrent vertigo, with a lifetime prevalence of 2.4%.^[5]^ The accepted pathology of BPPV is the “cupulolithiasis” or “heavy cupula” theory proposed by Schuknecht^[6]^ in 1962, which held that the most common form of BPPV occurs when otoliths from the macula of the utricle fall into the semicircular canal responding to the effect of gravity, but the cause of detachment remains obscure. Secondary BPPV can be caused by various inner ear diseases, head trauma and surgery, and the common inner ear diseases including SSNHL, Meniere disease, and vestibular neuritis.^[7–10]^ The pathogenesis of BPPV secondary to different inner ear diseases is also different.^[7]^ Previous studies have showed that the percentages of secondary BPPV in BPPV patients was in the range of 3% to 66%.^[7,8,11,12]^

For the therapy of SSNHL, steroid is the only drug considered to be most effective,^[13]^ which can be administered orally or intravenously. The role of the steroid may be to reduce inflammation and edema in the hearing organs.^[14]^ Some hypotheses^[15]^ of the pathogenesis of SSNHL include as viral infection, vascular compromise, autoimmune disease, inner ear pathology, so the combination of related drugs and steroids will be used in clinic. The incidence of BPPV secondary to SSNHL is about 5% to 19%.^[16,17]^

At present, the most effective treatment method for primary BPPV is manual reduction,^[18]^ whereas for SSNHL with BPPV, the effect of manual reduction is uncertain. Some scholars^[19,20]^ believe that the effect is the same as for primary BPPV, perhaps requiring repeated reduction more times, and some^[16]^ think that the effect is poor but reduction is still the best choice. Our experience found that the vertigo symptoms caused by BPPV secondary to SSNHL within 1 week of onset could not be resolved by reduction, but would be gradually relieved with the treatment of SSNHL, which was inconsistent with the research results of previous studies. Therefore, in this study, we aimed to providing guidance for the clinical treatment of patients with BPPV secondary to SSNHL within 1 week of onset. The following describes the process and results of this study.

## 2. Materials and methods

This randomized controlled trial was approved by the Medical Ethical Committee of the Hebei Provincial Eye Hospital (2020KY026). This was a randomized, prospective, parallel group, double-blinded study, and simple randomized sampling was done by lottery method. Ninety patients ---, who had been diagnosed BPPV with SSNHL within 1 week of onset and showed a normal MRI of the brain and the inner ear were recruited from Hebei Provincial Eye Hospital from June 2020 to December 2022, and 210 primary BPPV outpatients were admitted during the same period. Patients with cardiovascular and cerebrovascular diseases such as hypertension, diabetes, kidney disease, and immune system diseases were excluded. The patients with SSNHL were first examined for BPPV and then randomly divided into 2 groups: the medicine plus manual reduction group (abbreviated as the reduction group) and the medicine group. For the reduction group, the subjects were first received with reduction followed by a week of medication, while those in the medicine group were treated directly with medication for 1 week. The primary BPPV group were treated relevant reduction therapy according to the type of BPPV. We used the Epley method for posterior semicircular canal BPPV (psc-BPPV) and anterior semicircular canal BPPV (asc-BPPV), while for horizontal semicircular canal BPPV (hsc-BPPV), we used the Barbecue method. SSNHL is defined as a sensorineural hearing loss of ≥ 30 dB in 3 sequential frequencies within 3 days.^[15]^ BPPV is a temporary vertigo induced by moving the head to a specific position.^[4]^ The types of BPPV^[21,22]^ were determined by the direction of the nystagmus using the Dix-Hallpike test and Roll test. Psc-BPPV was diagnosed with upward rotation of the nystagmus with vertigo during the Dix-Hallpike test, and asc-BPPV was diagnosed with downward rotation. When the head moves from side to side in the roll test, we can see the same direction of the nystagmus as the head movement. The side with strong nystagmus is defined as the affected side, which can be diagnosed as hsc-BPPV. For manual reduction of psc-BPPV, we used the Epley method, and for hsc-BPPV, we used the Barbecue method.

The details of Epley is (take the left side for example): Head is placed over the end of the table, 45 degrees to the left, then rotated to 45 degrees right, then rotated until facing downward 135 degrees from supine position, and finally patient is brought to sitting position.^[23]^

The details of Barbecue is: The patient lie down with the affected ear facing downward; He or she is taken through a series of step-wise 90-degree turns away from the affected side until he returns to the original position. Holding each position for 10 to 30 seconds.^[24]^

The effect on the day after reduction is called immediate efficacy, and the effect of 7 days after reduction is called 7th-day efficacy. And the effect was divided into 3 grades: cure = the symptoms of nystagmus and vertigo all disappeared; improvement = the symptoms of nystagmus and/or vertigo were alleviated; ineffective = the nystagmus and vertigo remained unchanged or even worsened; effective rate = cure rate + improvement rate.

All of the patients with SSNHL accompanied by BPPV underwent bitheral caloric test and vestibular evoked myogenic potential (VEMP) test.

Bithermal caloric test using Type 1068 (GN OTOMETRICS A/S) was performed and the steps were: The patient was supine with the head elevated to a 30° angle and his/her ears were irrigated with 24°C of cool air and 50°C of warm air. Finally, canal paresis percentages were calculated by the Jongkees equation, and its value >25% indicates reduced function of the left or right horizontal semicircular canal.^[25]^

VEMP test includes cervical VEMP (cVEMP) and ocular VEMP (oVEMP), and we used Neuro - Audio (Made in Russia) to test it.

The measuring method of oVEMP was as follows: The patients was placed in a seated position with the head upright, and the eyes asked to look up 30° to 45° upward during the recording. The doctor attached electrodes to the patient according to the standard procedure.^[26,27]^ The resultant response consisted of a biphasic wave with an initial negative peak N1 and a subsequent positive peak P1. Asymmetry ratio is calculated from the magnitude values of P1 and N1, asymmetry ratio > 40% is abnormal.^[28]^

The measuring method of cVEMP was as follows: The patients was placed in a seated position and the head is tilted to the opposite side of the ear, and then attached electrodes according to the standard procedure.^[29]^ The results are interpreted with the same standard as oVEMP.

For the medicine, the subjects were given vasodilators (extract of Ginkgo biloba leaves injection 25 mL and betahistine hydrochloride injection 20 mg) and oral glucocorticoid (prednisone, 1 mg/kg/day for 3 days and maximum dose is 60 mg). SPSS22.0 statistical software (IBM, Armonk, NY) was used for data analysis. The chi-square test was used for comparison between the 2 groups, and *P* < .05 was considered significant.

## 3. Results

The primary BPPV group include 210 participants, 91 males and 119 females, aged 18 to 71 years. Of them, 140 were psc-BPPV, 50 were hsc-BPPV, 13 were asc-BPPV, and 7 were multiple semicircular canal BPPV (msc-BPPV) cases. The reduction group include 45 participants, 19 males and 26 females, aged 22 to 67 years, Among them, 23 were psc-BPPV, 19 were hsc-BPPV and 3 were msc-BPPV. And the medicine group include 45 patients, 20 males and 25 females, aged 21 to 67 years, of them, 22 were psc-BPPV, 19 were hsc-BPPV and 4 were msc-BPPV.

The bithermal caloric test of 90 patients with SSNHL showed that there were 80 patients with canal paresis values >25%, which meaned that the function of the horizontal semicircular canal on the deaf side was impaired.

About the VEMP in the SSNHL, there were 16 patients with abnormal results of both oVEMP and cVEMP, 29 with abnormal oVEMP results, and 15 with abnormal cVEMP results.

There were no significant differences in 7th-day efficacy of reduction for psc-BPPV patients between the medicine group and reduction group (*P* = .67, Table 1). There were no significant differences in 7th-day efficacy of reduction for hsc-BPPV and msc-BPPV patients between the medicine and reduction groups (*P* = 1.0, Table 2; *P* = 1.0, Table 3).

**Table 1:** Comparison of the 7th-day efficacy of reduction for psc-BPPV patients between the medicine group and reduction group.

Group	Cases	Clinical effect			Effective rate (%)
		Cure	Improvement	Ineffective	
Medicine group	22	20	2	0	100
Reduction group	23	20	3	0	100

Chi-square test: *P *= .673.

psc-BPPV = posterior semicircular canal BPPV.

**Table 2:** Comparison of the 7th-day efficacy of reduction for hsc-BPPV patients between the medicine group and reduction group.

Group	Cases	Clinical effect			Effective rate (%)
		Cure	Improvement	Ineffective	
Medicine group	19	16	3	0	100
Reduction group	19	17	2	0	100

Chi-square test: *P* = 1.

hsc-BPPV = horizontal semicircular canal BPPV.

**Table 3:** Comparison of the 7th-day efficacy of reduction for msc-BPPV patients between the medicine group and reduction group.

Group	Cases	Clinical effect			Effective rate (%)
		Cure	Improvement	Ineffective	
Medicine group	4	2	2	0	100
Reduction group	3	1	2	0	100

Chi-square test: *P* = 1.

msc-BPPV = multiple semicircular canal BPPV.

There were significant differences in immediate efficacy of reduction for psc-BPPV and hsc-BPPV patients between the the primary BPPV group and the reduction group (*P* *<* .001, Table 4; *P* *<* .001, Table 5). However, there were not significant differences for msc-BPPV patients (*P* = .08, Table 6). Finally, because asc-BPPV was not present in BPPV secondary to SSNHL, the statistics could not be made.

**Table 4:** Comparison of the immediate efficacy of reduction for psc-BPPV patients between the primary BPPV and the reduction group.

Group	Cases	Clinical effect			Effective rate (%)
		Cure	Improvement	Ineffective	
Primary BPPV	140	98	37	5	96.4
Reduction group	23	0	3	20	13.0

Chi-square test: *P *< .001.

BPPV = benign paroxysmal positional vertigo, psc-BPPV = posterior semicircular canal BPPV.

**Table 5:** Comparison of the immediate efficacy of reduction of hsc-BPPV patients between the primary BPPV and the reduction group.

Group	Cases	Clinical effect			Effective rate (%)
		Cure	Improvement	Ineffective	
Primary BPPV	50	26	18	6	88.0
Reduction group	19	0	2	17	10.5

Chi-square test: *P* < .001.

BPPV = benign paroxysmal positional vertigo, hsc-BPPV = horizontal semicircular canal BPPV.

**Table 6:** Comparison of the immediate efficacy of reduction of msc-BPPV patients between the primary BPPV and the reduction group.

Group	Cases	Clinical effect			Effective rate (%)
		Cure	Improvement	Ineffective	
Primary BPPV	7	0	7	0	100
Reduction group	3	0	0	3	0

Chi-square test: *P* *=* *.08.*

BPPV = benign paroxysmal positional vertigo, msc-BPPV = multiple semicircular canal BPPV.

## 4. Discussion

Our study found that the diseased side of BPPV secondary to SSNHL was consistent with that of deafness in the 90 enrolled patients. The most common type of BPPV was psc-BPPV, followed by hsc-BPPV, and msc-BPPV was rare.^[30,31]^ In addition, the most important result of our study, which is different from that of previous studies,^[16,19,20]^ is that regarding the treatment of BPPV secondary to SSNHL within 1 week of onset, we found that reduction therapy is not recommended, and medication is the first choice. That is because not only the effect of manual reduction is poor, but also patients suffer great pain during the process. The vertigo will be reduced significantly or even disappear after a week of medication for SSNHL. The analyses of the results of this study are as follows.

First, we found that the side of BPPV was consistent with the side of deafness in the patients with SSNHL accompanied by BPPV. This result is consistent with previous studies. SSNHL is a common ear disease. According to statistics, 28.91% of SSNHL patients experience dizziness or vertigo in China.^[32]^ BPPV is one of the causes of vertigo.^[20,33]^ Th cause of SSNHL accompanied BPPV may be the abnormal blood supply to the inner ear. Normally, the otoliths are attached to the utricle, and the blood comes from the labyrinthine artery, which is a slender artery with a diameter of 0.18 mm that has no collateral circulation.^[34]^ It is supposed that once abnormal supply of the labyrinthine artery occurs, the cochlea is often damaged, along with the vestibule, which then causes the otoliths to fall off.

The second conclusion of the study is about the types of BPPV. That is, regarding the composition in primary BPPV, psc-BPPV accounted for 66.7%, hsc-BPPV for 23.8%, asc-BPPV for 6.2%, and msc-BPPV for 3.0%. In the patients with SSNHL accompanied by BPPV, psc-BPPV accounted for 50.0%, hsc-BPPV for 42.2%, and msc-BPPV for 7.8%. We found that compared with primary BPPV, for BPPV secondary to SSNHL, the proportion of hsc-BPPV and msc-BPPV was significantly increased (*P* *<* .05, Table 7), but there were no cases of asc-BPPV. This is similar to previous research.^[35]^ The most recognized reason for psc-BPPV in primary BPPV is the action of gravity, which makes the otoliths fall into the semicircular canal. However, BPPV secondary to SSNHL is supposed to be caused by the insufficient blood supply from the inner ear, so the incidence of hsc-BPPV and msc-BPPV increases. There are 2 reasons for the absence of asc-BPPV. First, asc-BPPV is rarely seen overall, so it is more difficult to find in SSNHL with BPPV. Second, the clinical signs and symptoms are often atypical in asc-BPPV, so they were not selected in the study sample.

**Table 7:** Comparison of the proportion of each type of BPPV between the primary BPPV and the SSNHL with BPPV group.

Group	psc-BPPV	hsc-BPPV	msc-BPPV	asc-BPPV
Primary BPPV	66.7%	23.8%	3.0%	6.2%
SSNHL with BPPV	50.0%	42.2%	7.8%	0%
*P* value	.007	.001	.169	.035

Chi-square test: *P* < .05.

asc-BPPV = anterior semicircular canal BPPV, BPPV = benign paroxysmal positional vertigo, hsc-BPPV = horizontal semicircular canal BPPV, msc-BPPV = multiple semicircular canal BPPV, psc-BPPV = posterior semicircular canal BPPV, SSNHL = sudden sensorineural hearing loss.

The third and most important conclusion of the study is regarding the treatment of patients with BPPV secondary to SSNHL within 1 week of onset. We know the most effective treatment is manual reduction for primary BPPV, and our research shows that the immediate efficacy was 96.4% for psc-BPPV, 88% for hsc-BPPV, 100% for msc-PPV and 38.5% for asc-BPPV. However, it is different for SSNHL with BPPV, for which we found that 87.0% was ineffective for psc-BPPV, 89.5% for hsc-BPPV and 100% for msc-BPPV. Additionally, patients suffer greatly during the reduction process. They are prone to nausea, vomiting, palpitation, numbness of hands and feet, and other symptoms. If such patients are treated with medication instead of manual reduction for a week, the vertigo symptoms will also be reduced or even disappear. That is different from previous studies that displayed that manual reduction is just as effective for primary BPPV.^[16,19,20]^ The main reason for the different results is the design of the study, for which we selected patients within 1 week of onset. In addition, it may be related to the small samples in the previous studies. One reason for the poor effect of manual reduction may be that the pathogenesis of primary BPPV and secondary BPPV is different. The pathogenesis of primary BPPV remains unclear. There are the canalithiasis theory and eupulolithiasis theory at present; however, risk factors include age, mental stress, osteoporosis, insomnia, and hypertension.^[36,37]^ According to Schuknecht, BPPV is caused by otoconia detachment from the utricle, but the cause of detachment remains obscure, especially in primary BPPV.^[38,39]^ Whatever the explanation, the final result is that only the otoliths were detached, without damage to the semicircular canal. However, for patients with SSNHL and BPPV, because of the extensive ischemia of the inner ear, including the elliptic and semicircular canal, otoliths are detached from the utriculi, and the function of the semicircular canals is impaired, which might affect the movement of the otoliths. The evidences of vestibular impairments are the results of bithermal caloric test and VEMP test. According to our statistics, 88.9% of patients had abnormal function of the bithermal caloric test, and 66.7% had abnormal results of VEMP test, which suggested that the function of semicircular canal and otolithol on the affected side were impaired. Furthermore, there are other possibilities for the pathogenesis, such as inner ear bleeding.^[40]^ The vertigo in patients with SSNHL and BPPV might be caused by the erythrocyte fragments, not otoliths, moving into the endolymph after bleeding. Therefore, it is impossible for manual reduction of BPPV to have an effect. With the continuous absorption of fragments, the symptoms of vertigo will gradually be relieved. There is another hypotheses^[41]^that indicates that the vertigo in SSNHL accompanied by BPPV is due to the utricle and saccule being damaged by the inner ear ischemia, which cannot inhibit the excitability of the semicircular canal, resulting in nystagmus, which is similar to BPPV when the head moves. Because the otoliths are not detached in this process, the reduction has no effect, and the vertigo will be relieved with the recovery of the utricle and saccule function. Based on the above theories, the patients with BPPV caused by SSNHL were given medication instead of manual reduction.

For the treatment of SSNHL, the drugs are often similar despite the various possible causes.^[42]^ The drugs we used included extract of Ginkgo biloba leaves injection, betahistine hydrochloride injection and oral prednisone. The function of extract of Ginkgo biloba leaves injection is to remove excess free radicals, improve blood circulation, increase the supply of oxygen and glucose to ischemic tissue; and the role of betahistine hydrochloride injection is to increase the blood flow to the cochlea and vestibule and eliminate lymphoid edema,^[43]^ and thus cure vertigo; Glucocorticoid is a conventional drug for the treatment of SSNHL,^[44]^ although its pathogenesis is still unclear, the possible reasons are anti-inflammatory and immune effects, the regulation of inner ear electrolyte balance and the maintenance of microcirculation for inner ear. After 1 week of treatment of above drugs, the inner ear and vestibule circulation improved, and the symptoms of vertigo were alleviated.

Although we conducted a scientific study and obtained some meaningful results that are helpful to the clinic, there are still some questions to be answered, why is vestibular function not impaired in all patients, and why else and why is vestibular function not impaired in patients with effective reduction. We believe that we will make new discoveries in the future with continuous research and observation of larger samples.

## 5. Conclusion

We studied patients with SSNHL accompanied by BPPV within 1 week of onset and found that the onset side of BPPV is that same as that of SSNHL, and msc-BPPV was more common than primary BPPV, aside from psc-BPPV and hsc-BPPV. The new findings are that we used medicine treatment alone for BPPV secondary to SSNHL during the period, and manual reduction is not recommended. Reduction is not effective and may aggravate the patient’s suffering. The vertigo caused by BPPV will be relieved with SSNHL therapy.

## Acknowledgements

I would like to extend my deep gratitude to all those who have offered me a lot of help and support in the process of the research. First and foremost, my sincere thanks go to Dr. Wang, who has offered me numerous valuable comments and suggestions throughout my study. Without his painstaking teaching and insightful advice, the completion of this thesis would have been impossible. Also, I am grateful to my colleagues who give me a great deal of support and help in my daily life as well as study during the composition of this research. Finally, I really want to thank my husband and my children, for their love make me face the challenges of life and continually pursue my dream bravely.

## Author contributions

**Conceptualization:** Gui Fang Li.

**Data curation:** Gui Fang Li, Man Liu, Yan Zhuo Zhang, Yue Tang Wang, Lan Su, Ran Ran Liu.

**Formal analysis:** Man Liu, Yue Tang Wang.

**Methodology:** Gui Fang Li, Man Liu, Yan Zhuo Zhang, Lan Su, Ran Ran Liu.

**Project administration:** Gui Fang Li.

**Resources:** Lan Su, Ran Ran Liu.

**Software:** Yan Zhuo Zhang, Yue Tang Wang.

**Writing – original draft:** Gui Fang Li, Yue Tang Wang.

**Writing – review & editing:** Gui Fang Li.
